# Human embryonic stem cells and good manufacturing practice: Report of a 1- day workshop held at Stem Cell Biology Research Center, Yazd, 27^th^ April 2017

**Published:** 2017-06-01

**Authors:** Fatemeh Akyash, Fatemeh Sadeghian-Nodoushan, Somayyeh Sadat Tahajjodi, Habib Nikukar, Ehsan Farashahi Yazd, Mostafa Azimzadeh, Harry D. Moore, Behrouz Aflatoonian

**Affiliations:** 1 *Stem Cell Biology Research Center, Yazd Reproductive Sciences Institute, Shahid Sadoughi University of Medical Sciences, Yazd, Iran.*; 2 *Department of Advanced Medical Sciences and Technologies, School of Paramedicine, Shahid Sadoughi University of Medical Sciences, Yazd, Iran.*; 3 *Department of Reproductive Biology, School of Medicine, Shahid Sadoughi University of Medical Sciences, Yazd, Iran. *; 4 *Centre for Stem Cell Biology (CSCB), Department of Biomedical Sciences, The University of Sheffield, Western Bank, Alfred Denny Building, Sheffield S10 2TN, UK.*

**Keywords:** Cell Therapy, Good Manufacturing Practice, Human Embryonic Stem Cell, Stem Cells

## Abstract

This report explains briefly the minutes of a 1-day workshop entitled; “human embryonic stem cells (hESCs) and good manufacturing practice (GMP)” held by Stem Cell Biology Research Center based in Yazd Reproductive Sciences Institute at Shahid Sadoughi University of Medical Sciences, Yazd, Iran on 27^th^ April 2017. In this workshop, in addition to the practical sessions, Prof. Harry D. Moore from Centre for Stem Cell Biology, University of Sheffield, UK presented the challenges and the importance of the biotechnology of clinical-grade human embryonic stem cells from first derivation to robust defined culture for therapeutic applications.

The first successful report about the derivation and culture of the human embryonic stem cells (hESCs) from human pre-implantation stage embryos was published by Thomson *et al* in 1998 (1). These pluripotent cells have two unique properties; not only, they can be maintained and expanded unlimitedly as undifferentiated cells, but also they are able to differentiate into every adult cell types which derived from three embryonic germ layers (ectoderm, mesoderm and endoderm) and germ cells (2-4).These capabilities have attracted researcher’s attention to use these cells in cell-based therapeutic applications, tissue engineering and regenerative medicine. However, it should be considered that good manufacturing practice (GMP) is essential for production of cell products from hESCs and induced pluripotent stem cells (iPSCs)- for clinical applications in human (5). GMP is known as part of quality management which controls the quality standards of the products. Several necessities defined for GMP, including, trained and qualified personnel which should be aware of GMP principles, suitable location and well-designed building with appropriate equipment and facilities, traceable documentation, right storage and transport with protection from contamination, clear labeling for materials and equipment, quality control and quality assurance (6).

In this regard, Recently, 1-day workshop entitled “Human Embryonic Stem Cells and Good Manufacturing Practice” sponsored by the Stem Cell Biology Research Center, Yazd Reproductive Sciences Institute, in collaboration with Prof. Harry D. Moore from Centre for Stem Cell Biology, University of Sheffield, UK as a keynote speaker in the field was hosted by the Shahid Sadoughi University of Medical Sciences on 27^th^ April 2017. This workshop was divided into three sessions. During the workshop two types of cells were used in practical sessions. The first practical session was about culture and passage of Yazd human foreskin fibroblast cell line (YhFFs # 8, P14 and P17; Aflatoonian *et al*, unpublished data) as a feeder layer for hESCs and hands on YAZD3 hESCs (P127; culture and passage of the cells on the YhFFs; Aflatoonian *et al* unpublished data). Following, theoretical session was about challenges regarding the derivation of hESCs and their therapeutic applications in GMP. Finally, for sharing of participants’ thoughts and ideas about the field a scientific discussion panel was held with presence of Behrouz Aflatoonian (Ph.D.), Habib Nikukar (M.D., Ph.D.), Ehsan Farashahi-Yazd (Ph.D.), Mostafa Azimzadeh (Ph.D.), and Prof. Harry D. Moore (Ph.D.) as panel members. 

Practical session commenced with training the passage and culture of the YhFFs first by observing and then each of the attendees was allowed to passage one flask individually. For this technique, culture medium was removed from a tissue culture flask of YhFFs and the cells were washed with 1-2 ml Dulbecco’s phosphate-buffered saline (PBS; Biowest, without Ca^2+^, Mg^2+^). 2 ml of trypsin-EDTA was added and the flasks of YhFFs incubated at 37^o^C at 5% CO_2_ for 3 min. Flasks were tapped to detachment of the cells from the bottom of the flasks. Trypsin-EDTA was neutralised by adding 2 ml of Dulbecco’s modified Eagle’s Medium (DMEM) supplemented with 10% fetal bovine serum (FBS; all from Gibco, Grand Island, NY). The mixture was aspirated and transported to the conical tube to centrifugation at 1000 rpm for 5 min. The pellet was seeded into a tissue culture flask containing DMEM/10%FBS medium and incubated in the humidified atmosphere at 37^o^C at 5% CO_2_.

After coffee break, in another part of practical session, passage and culture of YAZD3 hESCs was taught. YAZD3 hESCs were co-cultured on mitotically inactivated YhFFs in HES medium. Then, they were treated with 1 mg/ml collagenase type IV (Gibco, Grand Island, NY) for 8-10 min and then dissociated from YhFFs by glass pipette which plated onto the new cultured YhFFs with microdrop culture system (3), then incubated in the humidified atmosphere at 37^o^C at 5% CO_2_. Afterwards, participants performed all practical steps by themselves under faculty member’s supervision. After a praying and lunch break, during the theoretical session, Prof. Harry D. Moore talked about the importance of GMP for therapeutic application of hESCs. 38 of clinical-grade hESC lines from approximately 50 cell lines around the world have been derived from various centers in the UK which molecular karyotype of 25 these cell lines were evaluated by whole-genome single nucleotide polymorphism (SNP) technique (7). Centre for Stem Cell Biology, University of Sheffield derived 14 master cell lines named MasterShef1-14 which expression profile of specific markers were examined for confirmation of pluripotency of cell lines. Three of these MasterShef lines (MasterShef 2; Female, MasterShef7 and 10; Male) are released to UK Stem Cell Bank (UKSCB) for future clinical applications (8). 

Remarkably, approval from several regulatory agencies such as Human Fertility and Embryology Authority (HFEA), Human Tissue Authority (HTA) and Medicines and Healthcare products Regulatory Agency (MHRA) is essential for the derivation process of hESCs and their use in cell based therapies in UK (9).
